# The name of the game: palaeoproteomics and radiocarbon dates further refine the presence and dispersal of caprines in eastern and southern Africa

**DOI:** 10.1098/rsos.231002

**Published:** 2023-11-22

**Authors:** Louise Le Meillour, Antoine Zazzo, Séverine Zirah, Olivier Tombret, Véronique Barriel, Kathryn W. Arthur, John W. Arthur, Jessie Cauliez, Louis Chaix, Matthew C. Curtis, Diane Gifford-Gonzalez, Imogen Gunn, Xavier Gutherz, Elisabeth Hildebrand, Lamya Khalidi, Marie Millet, Peter Mitchell, Jacqueline Studer, Emmanuelle Vila, Frido Welker, David Pleurdeau, Joséphine Lesur

**Affiliations:** ^1^ Unité Archéozoologie, Archéobotanique: Sociétés, Pratiques, Environnements (AASPE), Muséum national d'Histoire naturelle, CNRS, CP 56, 55 rue Buffon, 75005 Paris, France; ^2^ Unité Molécules de Communication et Adaptations des Micro-organismes (MCAM), Muséum national d'Histoire naturelle, CNRS, CP 54, 57 rue Cuvier, 75005 Paris, France; ^3^ Section for Molecular Ecology and Evolution, Globe Institute, Faculty of Health and Medical Sciences, University of Copenhagen, Øster Farimagsgade 5, 1353 København, Denmark; ^4^ Unité Histoire naturelle de l'Homme Préhistorique (HNHP), Muséum national d'Histoire naturelle, CNRS, UPVD, 1 rue René Panhard, 75013 Paris, France; ^5^ Centre de Recherche en Paléontologie – Paris (CR2P), Muséum national d'Histoire naturelle, CNRS, Sorbonne Université, CP 38, 8 rue Buffon, 75005 Paris, France; ^6^ Department of Anthropology, University of South Florida St. Petersburg, 140 7th Avenue South, St. Petersburg, FL 33713, USA; ^7^ Unité Travaux et Recherches Archéologiques sur les Cultures, les Espaces et les Sociétés (TRACES), CNRS, Université Toulouse Jean Jaurès, 5 allées Antonio Machado, 31058 Toulouse, France; ^8^ Département d'archéozoologie, Muséum d'histoire naturelle (MHNG), 1 route de Malagnou, 1208 Genève, Switzerland; ^9^ Anthropology Program, California State University Channel Islands, 1 University Drive, Camarillo, CA 93012, USA; ^10^ Department of Anthropology, University of California, Santa Cruz, Social Sciences 1 Faculty Svcs, 1156 High Street, Santa Cruz, CA 95064-1077, USA; ^11^ Museum of Archaeology and Anthropology, University of Cambridge, Downing Street, Cambridge CB2 3DZ, UK; ^12^ Unité Archéologie des Sociétés Méditerranéennes (ASM), CNRS, Université Montpellier III, Ministère de la Culture, INRAP, Montpellier, France; ^13^ Department of Anthropology, Stony Brook University, Stony Brook, NY, USA; ^14^ Unité Cultures et Environnements. Préhistoire, Antiquité, Moyen Age (CEPAM), Université Côte d'Azur, CNRS, 24 avenue des Diables Bleus, 06300 Nice, France; ^15^ Département des Antiquités Égyptiennes, Musée du Louvre, Paris, France; ^16^ School of Archaeology, University of Oxford, Oxford, OX1 3TG, United Kingdom and Rock Art Research Institute, University of the Witwatersrand, PO Wits 2050, Johannesburg, South Africa; ^17^ Unité Archéorient, Environnements et sociétés de l'Orient ancien, CNRS, Université de Lyon 2, Maison de l'Orient et de la Méditerranée, 7 rue Raulin, 69007 Lyon, France

**Keywords:** Africa, zooarchaeology, hunters–gatherers, pastoralism, palaeoproteomics, radiocarbon dating

## Abstract

We report the first large-scale palaeoproteomics research on eastern and southern African zooarchaeological samples, thereby refining our understanding of early caprine (sheep and goat) pastoralism in Africa. Assessing caprine introductions is a complicated task because of their skeletal similarity to endemic wild bovid species and the sparse and fragmentary state of relevant archaeological remains. Palaeoproteomics has previously proved effective in clarifying species attributions in African zooarchaeological materials, but few comparative protein sequences of wild bovid species have been available. Using newly generated type I collagen sequences for wild species, as well as previously published sequences, we assess species attributions for elements originally identified as caprine or ‘unidentifiable bovid’ from 17 eastern and southern African sites that span seven millennia. We identified over 70% of the archaeological remains and the direct radiocarbon dating of domesticate specimens allows refinement of the chronology of caprine presence in both African regions. These results thus confirm earlier occurrences in eastern Africa and the systematic association of domesticated caprines with wild bovids at all archaeological sites. The combined biomolecular approach highlights repeatability and accuracy of the methods for conclusive contribution in species attribution of archaeological remains in dry African environments.

## 1. Introduction

The spread of domestic caprines throughout Africa is increasingly understood as a complex process. Although caprines appear to have arrived in far northern east Africa as part of a ‘package’ brought by migrating pastoralists [1,2], the introduction of domesticated animals into hunter–gatherer economies there and elsewhere in sub-Saharan Africa appears to have occurred gradually, as documented for other parts of the world [3]. Documenting the shift leading to herding is particularly crucial in African archaeological contexts, where introduction of domesticated animals might not have led to a complete cessation of hunting wild game [4–6]. Archaeological documentation of this shift depends upon reliable distinguishing between domestic and wild bovids. The origins of domestic sheep (*Ovis aries*) and goats (*Capra hircus*) are in southwest Asia [7–9]. The oldest African evidence of goat remains is from 7000–6800 BP at Haua Fteah, Libya [10], although these dates were criticized by the authors themselves, and at Sodmein Cave in eastern Egypt [11]; whereas the oldest sheep remains come from the Egyptian site of Merimde dated to 6000 BP [12,13]. In Kenya, the presence of caprines dates to *ca* 5000 cal. BP in the Lake Turkana basin [14,15], corresponding to the end of the African Humid Period, 14 800–5500 years BP [16]. Caprines later spread south, reaching southern Africa around 2000 BP [17,18]. With divergent behaviour and ecology, sheep and goats adapt differently to their environments. Moreover, at least two distinct breeds of each species exist in their southern ranges, suggesting several introductions [19,20]. Caprines probably spread from eastern to southern Africa along a ‘tsetse-free’ corridor from Kenya and southern Tanzania through Zambia to Botswana [21,22]. Genomic similarities of sheep and goat breeds reinforce the hypothesis of substantial contacts between the two regions [20,23]. Although tracing the routes taken by the first caprines on the continent remains a challenge, genomic and linguistic evidence argues in favour of stronger relations between eastern and southern caprine and human populations than between those in other areas, such as western Africa [24–30].

The close morphological similarities of sheep and goats, together with the fragmentary condition of archaeological skeletal remains, often prevent distinction of the two species. Such fragmentary remains are frequently recorded as ‘sheep/goat’ or ‘caprines’ in zooarchaeological analyses [31,32]. Further, skeletal specimens of autochthonous antelopes may readily be confused with those of caprines [18,33–36]. It is thus common for zooarchaeologists to place less identifiable specimens of similarly sized antelopes and domestic caprines in Bovid Size Class II [37]. Furthermore, Africa hosts the largest concentration of bovid species on the planet [38,39]. Within the Bovidae, eight subfamilies are reported, 50 genera, over 100 species and even more subspecies [40]. The taxonomy of the Bovidae is complex as it groups subfamilies initially defined by morphological features, some of which have been reclassified by molecular analyses. Depending on the study, the subfamilies Aepycerotinae, Hippotraginae and Reduncinae—sometimes even some species of Caprinae—are included in the Antilopinae subfamily, a sister group of Bovinae, which includes cattle and spiral-horned antelopes, such as lesser and greater kudu species, *Tragelaphus* spp. [41]. Based on maximum-likelihood results on mitochondrial DNA, Hassanin *et al.* defined four generic subgroups within the Antilopinae: Procaprina, Ourebina, Raphicerina and Antilopina, although no consensus has been found [42]. Chen *et al.*, on the contrary, based on full genome analyses, kept the following subfamilies within Bovidae: Aepycerotinae, Antilopinae, Bovinae, Caprinae, Hippotraginae and Reduncinae [43].

In this challenging research landscape, biomolecular methods can help distinguish archaeological remains of wild bovids from those of livestock. But the arid climates typical of much of Africa limit recovery of DNA [44]. However, while DNA is often degraded, other biomolecules, especially proteins, are more persistent in arid environments and offer valuable taxonomic information [45]. Intrinsically embedded in a mineral matrix composed of hydroxyapatite crystals [46], fibrils of type I collagen (COL1) persist longer than other molecules [47], and are often the only proteins recovered from remains in arid environments. Most palaeoproteomics studies conducted so far on African materials have focused on the application of peptide mass fingerprinting, or zooarchaeology by mass spectrometry (ZooMS) [48–53]. The method has, for instance, proven effective in tracing the ivory trade in South Africa [51], taxonomically identifying bone tools [50,52] or cross-validating caprine identification at Luxmanda, Tanzania [49]. Recently, both shotgun palaeoproteomics and ZooMS have been used to identify sheep and goat remains from both eastern and southern Africa independently [34,48,54], confirming the earliest presence of sheep in South Africa at Spoegrivier around 2000 cal. BP [18]. At the time the present work was initiated, type I collagen sequences had not been generated for the majority of African wild bovids, with the only protein sequences in international databases being those of cattle (*Bos taurus*), zebu (*Bos indicus*), wild yak (*Bos mutus*), a hybrid of *B. taurus* x *B. indicus*, chiru (*Pantholops hodgsonii* until recently), sheep (*O. aries*) and goat (*C. hircus*).

We thus report the first large-scale study of archaeological remains of putative caprines from eastern to southernmost Africa using biomolecular methods. First, we present an exploitable COL1 reference set for zooarchaeological research that is especially relevant to the diffusion of domestic caprines in Africa. We establish by high-throughput tandem mass spectrometry the COL1 sequences from nine species of wild bovids from Africa that might be confused with domesticated caprines in archaeological contexts. Combined with published COL1 sequences [39], they constitute a large reference library for identification of archaeological remains of African bovids. Secondly, we use these findings to undertake taxonomic identification of 114 archaeological specimens from 17 archaeological sites in eastern and southern Africa that have previously been morphologically identified as putative caprines or Brain's Size Class II bovids. Finally, direct radiocarbon dates were obtained for the sheep and goat remains identified by proteomics. These analyses confirm the presence of domesticates at nine of the 17 sites, enabling us to further discuss the potential of these paired methods for enriching research on the introduction of domestic caprines into Holocene African subsistence economies.

## 2. Materials

### 2.1. Modern material

Previously, very few species of Bovidae type I collagen were available in public repositories (such as the National Center for Biotechnology Information (NCBI) or UniProt, commonly used in proteomics). In 2021, Janzen *et al.* [39] published COL1 references for a large number of African wild bovids. The proposed sequences are either translated from genomic data or obtained from one individual analysed through liquid chromatography–tandem mass spectrometry (LC-MS/MS) [39]. To avoid redundancy, species published in the Janzen *et al*. paper are not reported in the main text of this manuscript, although some overlap of species exists due to concurrent projects (for more details, see electronic supplementary material, table S1).

**Figure 1.**
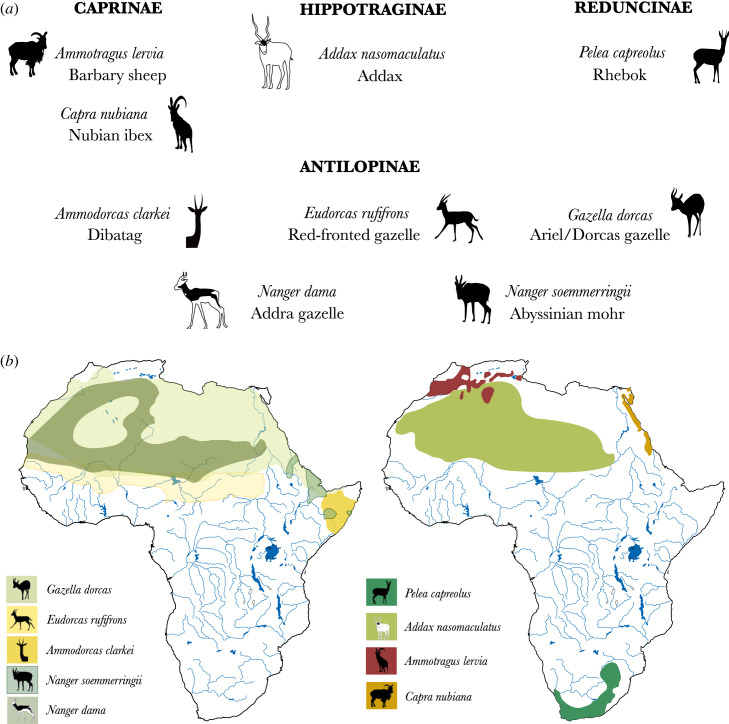
Nine species of African wild bovids sampled from the Muséum national d’Histoire naturelle (MNHN) collections for which new COL1 sequences are presented in this paper. (*a*) Sampled species are presented by subfamily and using both their scientific and vernacular names. (*b*) Current geographical distributions of all nine species in Africa are represented (after [38]).

Wild bovid species in this study were selected based on their potential for skeletal confusion with domesticated caprines, and their biogeographical distributions, following a long corridor from northeastern to southern Africa via the Horn of Africa (figure 1). The latter is hypothesized to have been one initial route taken by the first herders in diffusing domestic sheep and goat across the sub-Saharan regions of the continent [7,17,34,55–57]. We sampled nine wild antelope species from four bovid subfamilies: Antilopinae, Caprinae, Hippotraginae and Reduncinae, all of which live in the vicinity of the eastern and southern archaeological African sites (figure 1). We sampled dental roots, or bone when teeth were unavailable. Two individuals of each species were sampled from the *Collections d'Anatomie Comparée* of the Muséum national d'Histoire naturelle (MNHN) in Paris, France (electronic supplementary material, table S1). One exception was *Ammodorcas clarkei*, of which only one specimen was available. The entire species and specimens sampled from museum collections can be found in electronic supplementary material, table S1, which also includes the nine species for which we present new COL1 de novo reference sequences (figure 1).

### 2.2. Archaeological faunal remains

**Figure 2.**
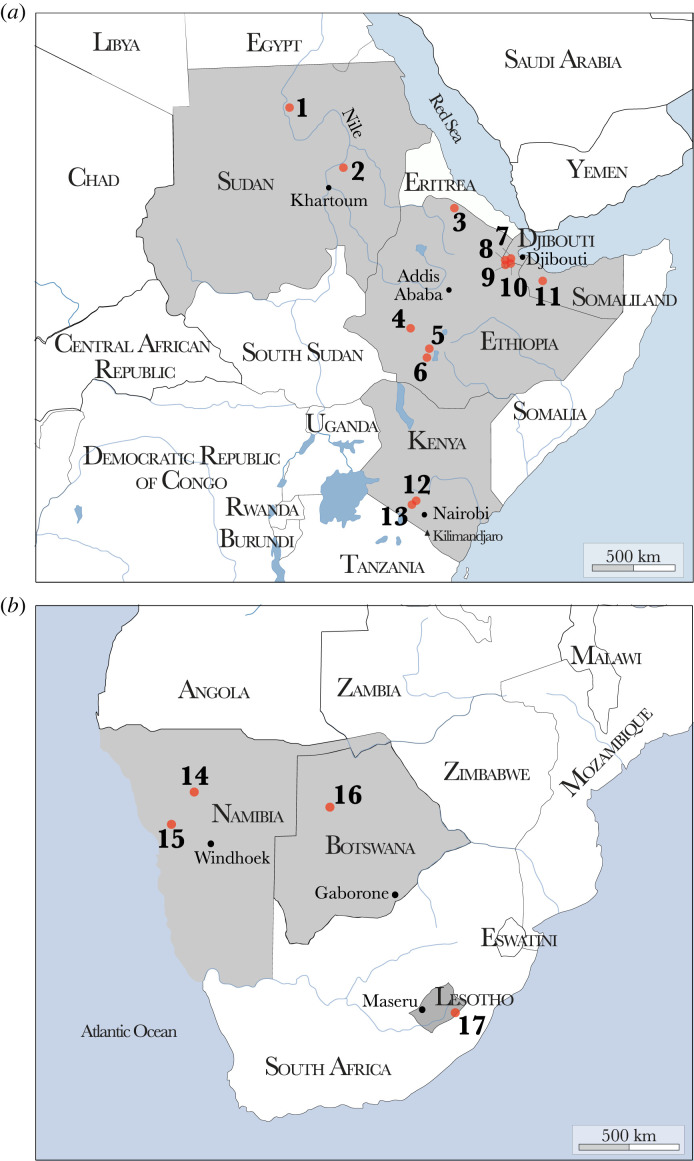
Locations of the archaeological sites from which the material of this study originates in eastern (*a*) and southern Africa (*b*). Numbers on both (*a*) and (*b*) maps correspond to the following sites: 1, Kerma; 2, Muweis; 3, Wakarida; 4, Kumali; 5, Mota Cave; 6, Garu; 7, Asa Koma; 8, Kurub; 9, Hedaito le Dora; 10, Wakrita; 11, Laas Geel; 12, Prolonged Drift; 13, Vaave Makonge; 14, Geduld; 15, Leopard Cave; 16, Toteng and 17, Melikane.

We chose 17 archaeological sites distributed between eastern and southern Africa to test the hypothesis of past connections between the two regions in the spread of domesticated sheep and goats. The selected faunal specimens included in our study presented morphological characteristics of domesticated caprines or were classified by the zooarchaeologists as ‘unidentified bovids’. Except for two of the archaeological sites, namely Toteng (no. 16 on figure 2*b*, [58]) and Leopard Cave (no. 15, figure 2*b*, [34]), none of the faunal remains from these sites have previously been subject to palaeoproteomics analyses. It is worth mentioning that the site of Mota Cave (no. 4, figure 2*a*) has yielded the burial of a human male, directly dated from 4500 years ago, whose genetic material indicates admixture between Eurasian and eastern African populations, indicating rather good biomolecular preservation at the site [59].

A hundred and fourteen remains from the 17 Holocene archaeological sites were sampled (electronic supplementary material, table S2). Seventeen specimens come from the sites of Muweis and Kerma in Sudan [60]; 48 from Wakarida [61], Mota Cave [62], Kumali [63], Garu [64], Kurub 7 and the Kurub Bahari plain [65], Asa Koma [66], Wakrita [67], Hedaito le Dora, Laas Geel [68], respectively, in Ethiopia, Djibouti and Somaliland; 11 from Prolonged Drift [69] and Vaave Makonge [70] in Kenya for eastern Africa. Southern African sites included fifty-three specimens from Geduld [71] and Leopard Cave [72] in Namibia; Toteng 1 and 3 [73] in Botswana and Melikane in Lesotho [74]. An extensive description of each archaeological site can be found in electronic supplementary material, data S1 and information 1 and their locations in eastern and southern Africa are provided in figure 2*a* and *b*, respectively.

## 3. Methods

### 3.1. ‘Modern’ and archaeological samples protein extraction

Both reference specimen samples and archaeological samples underwent the same protocol for protein extraction. Briefly, 10–20 mg of bone or tooth powder were sampled using an ethanol-cleaned diamond drill (electronic supplementary material, table S2). The archaeological specimens from Wakarida were mandibles with embedded teeth and thus both bone and tooth roots were sampled to assess the best tissue for protein extraction. For these, we obtained one bone sample and one dentine sample, for which organic preservation was assessed using the method described in [75]. Only the best-preserved sample according to the threshold discussed in [58] is presented here (electronic supplementary material, table S2).

After sampling, bone powders were placed in protein LoBind 2 ml tubes (Eppendorf, Germany). We followed the protocol for African remains, which is appropriate for the extraction and characterization of proteins from remains recovered from arid environments [34]. Bone or tooth powders were decalcified in a neutral pH buffer (tris(hydroxymethyl)aminomethane (Tris) 0.05 M and ethylenediaminetetraacetic acid (EDTA) 0.5 M, final pH 7.4, Sigma Aldrich, Germany) for 1–8 days, depending on the sample. Solutions were first replaced every half day, then every 24 h. When completely decalcified (i.e. until only a collagen ‘phantom’ remained), the pellets were rinsed five times with milliQ water. The extracted proteins were solubilized in 50 mM ammonium bicarbonate (ABC, pH 8, Sigma Aldrich) for 3 h at 65°C. Solutions were then centrifuged for 10 min at 3000*g* and collected into new microtubes. Extracted proteins were finally reduced with dithiothreitol (DTT, final concentration 10 mM, 20 min, 56°C, 350 r.p.m.), alkylated with iodoacetamide (final concentration 10 mM, 30 min, in the dark, at room temperature) and digested using trypsin (0.01 µg µl^–1^, Trypsin Gold, Promega). Finally, samples were transferred into clean vials and kept at −20°C until mass spectrometry analyses.

### 3.2. Mass spectrometry: ultra-high-performance liquid chromatography–tandem mass spectrometry

Digested protein extracts were analysed independently by ultra-high-performance liquid chromatography–tandem mass spectrometry (UHPLC-MS/MS) using a workflow described previously [34]. Separation was performed on an Ultimate 3000-RSLC system (Thermo Fisher Scientific) with a RSLC Polar Advantage II Acclaim column (2.1 × 100 mm, 120 Å, 2.2 µm) using a flow rate of 300 µl min^–1^ and mobile phase gradient of A: H_2_O + formic acid (FA) 0.1% and B: acetonitrile + FA 0.08%. We used a high-resolution ESI-Q-TOF mass spectrometer (Maxis II ETD, Bruker Daltonics) in positive mode and data-dependent auto-MS/MS mode on the *m/z* range 200–2200. MS/MS spectra were generated using collision-induced dissociation by selection of ions with charge states between 2^+^ and 5^+^ and on *m/z* range 300–2200. Calibration was carried out for each run with sodium formate clusters and experimental blanks analysed every five samples for each of the runs with modern references and archaeological samples.

### 3.3. Type 1 collagen sequences de novo reconstruction

The raw data were converted to .mgf using the mass spectrometer manufacturer software, DataAnalysis (v. 4.4, Bruker Daltonics). The sequences were reconstructed using a database-assisted tool using the Byonic workflow C57 (Byologic, Protein Metrics). The restricted database used for the search contained COL1A1 and COL1A2 sequences of the following Bovidae species: *Bos taurus* (P02453 and P02465), *B. mutus* (L8IV51 and L8HQF7), *B. indicus* (A0A4W2FAL4 and A0A4W2FTM9), *C. hircus* (A0A452FHU9 and A0A452G3V6), *O. aries* (W5P481 and W5NTT7) and *Pantholops hogsonii* (XM_005964647.1 and XM_005985683.1). The referenced sequences can be found on either NCBI or UniProt public repositories and correspond to entire translated protein sequences, including signal peptide and tropocollagen. Then, in order to reconstruct only the secreted protein sequences, we excluded these parts of the sequence from further analyses. When referring to amino acid positions, we use the sheep reference protein sequences. The alpha 1 chain starts (first position in electronic supplementary material, information 2) at position 170 (Q) and ends at position 1229 (K) in the UniProt reference (W5P481). Similarly, the alpha 2 chain starts at position 80 (Q) and ends at position 1117 (A) in the UniProt reference (W5NTT7). The software parameters were set to 1% false discovery rate (FDR), and post-translational modifications were as follows: we allowed three tryptic missed cleavages; carbamidomethylation of cysteines was set as fixed modification; deamidation of N and Q, Gln to pyro-Glu (N term Q), phosphorylation of S and T and oxidation of M and P were set as variables modifications, with a maximum of five modifications allowed for one peptide. In the Byonics workflow, proline oxidations (HyP) were considered as ‘common modification’ and every amino acid substitution was included in the ‘rare modification’ list. Once results were obtained, peptides with potential single amino acid polymorphisms (SAPs) were manually assessed by verifying MS/MS spectra with at least two peptide spectral matches (PSMs) in each of the two samples per species. For each species-specific peptide detected in a sample, we verified that the alternate sequence was absent by assessing PSMs manually (electronic supplementary material, data S2). We confirmed every sequence by aligning them using the Geneious Prime software (v. 2023.0.4) before building the final fasta file (electronic supplementary material, information 2 and Dryad data associated with the paper).

### 3.4. Archaeological samples identification using type I collagen sequences

Since Janzen *et al*. [39] published COL1 sequences of some of the species we had initially included in our dataset, the ‘overlapping’ species sequences were excluded from this paper. We hereby only present data for nine species (compared with the 19 initially sampled for reference purposes): *Ammodorcas clarkei*, *Eudorcas rufifrons*, *Gazella dorcas*, *Nanger dama*, *N. soemmeringi*, *Ammotragus lervia*, *Capra nubiana*, *Addax nasomaculatus* and *Pelea capreolus*. All other species COL1 sequences used for reference were taken from Janzen *et al*. All archaeological samples were searched using the MaxQuant software (v. 2.1.3.0, [76]) against the updated database of bovid species (electronic supplementary material), and using the following parameters: trypsin allowed missed cleavages was set on 3; carbamidomethylation of cysteines was set as fixed modification, and deamidation (N,Q), Gln to pyro-Glu (N term Q), phosphorylation (S, T) and oxidation (M, P) as variable modifications; mass tolerances were set to 10 ppm for precursor and 0.02 Da for fragment ions; all other parameters were left as default. We considered species identification confident if at least two razor and unique peptides from non-overlapping parts of the sequence were covered by inspecting the evidence file provided after the MaxQuant search (electronic supplementary material, data S3 and S4). In addition, we performed manual assessment of the species-specific peptides spectra.

### 3.5. Direct dating of remains identified as domesticated caprines

The remains molecularly identified as either sheep or goat were directly dated by radiocarbon analyses. Approximately 500 mg of bone powder was resampled and sieved to keep only the 0.3–0.7 mm fractions. Then, the bone powder was immersed in 1 M HCl for 20 min under continuous stirring and the acid-insoluble residues separated from the solution by centrifugation and finally rinsed with Milli-Q water using a vortex. Pellets were immersed again in 0.1 M NaOH, for 30 min under stirring and solution was changed after 10 min. After centrifugation, they were rinsed again, and the alkali-insoluble residues underwent another immersion into 0.01 M HCl, and solubilization was realized overnight at 95°C. The solubilized samples were filtered on mixed cellulose ester membranes (MF-Millipore, 5.0 µm pore size, Fisher Scientific, Illkirch, France) before freeze-drying and collection for further analysis. One sample, namely GrJi_165 was very small and its preparation was more delicate. Hence, extraction and graphitization were realized on the ^14^C laboratory lab lines in the MNHN. For all the other samples, graphitization was realized using the automated AGE 3 graphitization unit and the accelerator mass spectrometry (AMS) measurements using the compact AMS ECHoMICADAS at the Laboratoire des Sciences du Climat et de l'Environnement (LSCE, CEA, CNRS, UVSQ, Saclay, France).

## 4. Results

### 4.1. Type I collagen sequences of African wild bovids

The LC-MS/MS data provided around 1000 acquired MS2 spectra per reference sample. One sample only, namely WALL_3 (of *Litocranius walleri*) gave no usable results. Database-assisted de novo sequencing from LC-MS/MS data permitted the reconstruction of the COL1A1 and COL1A2 sequences from the 19 species of interest (electronic supplementary material, table S1), of which only nine are presented here (see Methods). All the other 10 species presented the same sequences as the ones available from Janzen *et al*. [39]. A total of 18 sequences with 100% sequence coverage were obtained for all nine species. Within the referenced species, we only observed 11 amino acid substitutions, with a predominance of alanine (A), threonine (T), valine (V), serine (S) and methionine (M) being the varying amino acids. Most of the observed differences show only one SAP resulting in only two variant peptides (electronic supplementary material, data S2). Interestingly, four peptides of COL1A2 show SAPs at between two and eight positions. The two markers classically employed to distinguish sheep and goat, **X**GEVGPPGPPGPAGEK from COL1A1 (X = A for *O. aries*, X = P for *C. hircus*) and GPSGEPGTAGPPGTPGPQG**Y**LG**Z**PGFLGLPGSR from COL1A2 (Y = L and Z = A for *O. aries*, Y = F and Z = P for *C. hircus*), are shared with the other phyla, as previously reported [34]; the ‘sheep version’ COL1A2 peptide is shared with most of Antilopinae, whereas the ‘goat version’ is present only in the *Capra* genus.

The alpha 1 chain of type I collagen shows six varying peptides (electronic supplementary material, data S2). Only one is specific to one species, *Oreotragus oreotragus*, 10 … S**A**GISVPGPMGPSGPR … 25. The peptide marker 721 … VGPPGPSGNAGPPGPPGP**V**GK … 741 discriminates the Tragelaphini tribe from all other species, while the sequence 980 … GPPGSAG**S**PGK … 990 is found both in Tragelaphini and the genus *Bos* (and in *Neotragus pygmaeus*—Janzen's data). The Caprinae share most of the peptide marker sequences, except for one diagnostic peptide which can be of use for the identification of the wild Caprinae species *C. nubiana* 161 … GNDGATGAAGPPGPTGPAGPPGFPGA**M**GAK … 199 (M substituted to V in *A. lervia*, *C. hircus* and *O. aries*).

Analyses of alpha 2 chain sequences indicate 13 peptides where SAPs are observed between the different species (electronic supplementary material, data S2). Four of them are species-specific, namely 333 … AG**G**MGPAGSR … 342 to *Addax nasomaculatus*, 352 … GP**S**GDSGR … 369 to *Oreotragus oreotragus*, 613 … GEAG**A**AGPAGPAGPR … 627 to *Antidorcas marsupialis* (the latter already identified, [34,39]). Peptide 667 … GENGPVGP**X**GP**Y**GAAGPSGPNGPPGP**Z**GSR … 696 is only present in Reduncinae (X = S, Y = V and Z = A), *Tragelaphus* sp. (X = T, Y = V and Z = P) and springbok (X = T, Y = A and Z = A). Specific to the subfamily Caprinae are the two peptides 750 … **T**GE**P**GA**A**GPPGF**V**GEK … 765 and 894 … HG**S**RGEPGP**V**GAVGPAGAVGPR … 927. One peptide shows five variant sequences resulting from substitutions at two positions, 868 … GYPGNAGPVGA**X**GAPGPQGPVGP**Z**GK … 896 (where X = A or V and Z = V, T, I/L or A), of which two are isomeric, GYPGNAGPVGA**A**GAPGPQGPVGP**V**GK reported for *B. taurus*, *B. mutus*, *O. oreotragus, R. campestris,* Tragelaphini, Caprinae, Hippotraginae and GYPGNAGPVGA**V**GAPGPQGPVGP**A**GK detected for the other species of Antilopinae. The latter was clearly differentiated by MS/MS in our sample of *A. clarkei* (electronic supplementary material, information 3).

### 4.2. Species identification of archaeological remains

Proteins were not preserved in five out of the 17 archaeological sites: namely Kumali, Asa Koma, Hedaito le Dora, Wakrita and Geduld. This prevents us from proposing molecular species identification of the faunal specimens from these sites. Four other sites present mixed results, with some but not all samples enabling identification: Kerma (3 of 9 samples were identified), Muweis (2 of 8), Mota Cave (2 of 4) and Laas Geel (2 of 3). For the remaining eight sites, taxonomic identifications were possible for all remains.

Based on the newly reconstructed African wild bovids COL1 sequences, along with the additional sequences available in the literature, we propose genus or species attributions for 86 of the 114 archaeological specimens, or 75.4% of the specimens examined in this study (table 1; electronic supplementary material, data S3 and S4). Based on the sequence homology observed for some species (e.g. within genus *Gazella*), some of the attributions presented here remain at the genus level, whereas for other specimens, taxonomic attributions are at the family level.

**Table 1.** Molecular identifications for all specimens included in the study. Samples are described by country, archaeological sites and molecular identifications are alongside the proposed morphological ones. ‘no result’ indicates that the sample did not contain enough preserved proteins to allow identification. Samples with * indicate that palaeoproteomics results did not allow to distinguish between two or more species belonging to the same subfamily (here Reduncinae). Given species distribution from Kingdon [38], only one species can be found in the area of the archaeological site and is thus reported in the table.

archaeological site	country	Lab_code	morphological identification	molecular identification (proteomics)
Kerma	Sudan	KER_99	*Capra hircus*	no result
		KER_100	*Capra hircus*	no result
		KER_102	*Ovis aries*	no result
		KER_103	cf. *Ovis*	no result
		KER_105	*Ovis aries*	*Ovis aries*
		KER_106	*Ovis aries*	*Ovis aries*
		KER_107	*Capra hircus*	Bovidae
		KER_108	*Capra hircus*	no result
		KER_109	*Capra hircus*	no result
Mouweis		MOW_45	small bovid	no result
		MOW_46	small bovid	no result
		MOW_47	small bovid	no result
		MOW_48	caprine	Bovidae
		MOW_49	bovid	Bovidae
		MOW_50	caprine	no result
		MOW_51	caprine	no result
		MOW_52	caprine	no result
Garu	Ethiopia	GAR_90	caprine	*Ovis aries*
		GAR_91	caprine	*Capra hircus*
		GAR_92	caprine	*Ovis aries*
		GAR_93	caprine	*Capra hircus*
Kumali		KUM_82	small bovid	no result
Kurub 7		KUR_56	caprine	no result
		KUR_57	caprine	no result
		KUR_59	caprine	no result
		KUR_60	caprine cf. *Capra*	no result
Kurub Bahari Plain		KUR_58	small bovid (?)	*Ovis aries*
Mota Cave		MOT_94	caprine	no result
		MOT_95	caprine	*Nanger* sp.
		MOT_96	caprine	no result
		MOT_97	caprine	Redunca redunca*
Wakarida		WAK_30	cf. *Capra*	*Ovis aries*
		WAK_32	cf. *Capra*	*Capra hircus*
		WAK_34	cf. *Capra*	*Capra hircus*
		WAK_36	cf. *Capra*	*Capra hircus*
		WAK_37	cf. *Ovis*	*Ovis aries*
		WAK_39	caprine	*Capra hircus*
		WAK_41	cf. *Ovis*	*Ovis aries*
		WAK_42	cf. *Capra*	*Ovis aries*
		WAK_44	cf. *Capra*	*Capra hircus*
Asa Koma	Republic of Djibouti	ASK_27	small bovid (?)	no result
		ASK_28	caprine	no result
Hedaito le Dora		HDL_68	caprine	no result
		HDL_69	caprine	no result
		HDL_70	small bovid	no result
		HDL_71	small bovid	no result
		HDL_72	caprine	no result
		HDL_73	caprine	no result
Wakrita		WAT_53	caprine	no result
		WAT_54	caprine	no result
		WAT_55	caprine	no result
Laas Geel	Somaliland	LG_61	small bovid/caprine	*Ovis aries*
		LG_62	small bovid/caprine	*Ovis aries*
		LG_63	small bovid/caprine	no result
Prolonged Drift (GrJi1)	Kenya	GrJi_165	small bovid	*Ovis aries*
		GrJi_166	*Nanger granti* or *Aepyceros*	*Nanger granti*
		GrJi_167	cf. *G. thomsoni*	*Aepyceros melampus*
		GrJi_168	medium bovid	*Ammodorcas clarkei*
		GrJi_169	small bovid cf. *G. thomsoni*	*Aepyceros melampus*
		GrJi_170	cf. *G. thomsoni*	Redunca redunca*
		GrJi_171	*Aepyceros? N. granti?*	*Aepyceros melampus*
Vaave Makonge (GvJm44)		GvJm_64	caprine	*Capra hircus*
		GvJm_65	caprine	*Capra hircus*
		GvJm_66	cf. *Capra*	*Ovis aries*
		GvJm_67	caprine	*Capra hircus*
Melikane	Lesotho	MLK_144	size 3 bovid	Pelea capreolus*
		MLK_145	caprine (?)	Pelea capreolus*
		MLK_146	size 3 bovid	Pelea capreolus*
		MLK_147	size 3 bovid	Pelea capreolus*
		MLK_148	size 3 bovid	Pelea capreolus*
		MLK_149	size 3 bovid	Pelea capreolus*
		MLK_150	caprine (?)	Pelea capreolus*
		MLK_151	size 3 bovid	Pelea capreolus*
		MLK_152	size 3 bovid	Pelea capreolus*
		MLK_153	size 3 bovid	Pelea capreolus*
		MLK_154	size 3 bovid	Pelea capreolus*
		MLK_155	size 3 bovid	Pelea capreolus*
		MLK_156	saprine (?)	*Oreotragus oreotragus*
		MLK_157	size 3 bovid	Pelea capreolus*
		MLK_158	size 3 bovid	Pelea capreolus*
		MLK_159	size 3 bovid	Pelea capreolus*
		MLK_160	*Ovis aries*	Pelea capreolus*
		MLK_161	*Ovis aries*	Pelea capreolus*
		MLK_162	*Ovis aries*	Pelea capreolus*
Geduld	Namibia	GE_81	*Ovis aries*	no result
Toteng		TOT_14	size 2/3 bovid	*Ovis aries*
Leopard Cave		LC_113	caprine	*Antidorcas marsupialis*
		LC_114	size 2/3 bovid	*Ovis aries*
		LC_115	size 2/3 bovid	*Ovis aries*
		LC_116	size 2/3 bovid	*Ovis aries*
		LC_117	size 2/3 bovid	*Ovis aries*
		LC_118	size 2/3 bovid	*Ovis aries*
		LC_119	size 2/3 bovid	*Ovis aries*
		LC_120	size 2/3 bovid	*Ovis aries*
		LC_121	size 2/3 bovid	*Ovis aries*
		LC_122	size 2/3 bovid	*Ovis aries*
		LC_124	size 2/3 bovid	*Ovis aries*
		LC_125	caprine	*Ovis aries*
		LC_126	caprine	*Ovis aries*
		LC_127	caprine	*Ovis aries*
		LC_128	caprine	*Ovis aries*
		LC_129	caprine	*Ovis aries*
		LC_130	caprine	*Ovis aries*
		LC_131	*Ovis aries*	*Ovis aries*
		LC_132	*Antidorcas/Aepyceros*	*Ovis aries*
		LC_133	*Antidorcas/Aepyceros*	*Ovis aries*
		LC_134	*Antidorcas/Aepyceros*	*Aepyceros melampus*
		LC_135	caprine	*Ovis aries*
		LC_136	*Antidorcas/Aepyceros*	*Ovis aries*
		LC_137	*Antidorcas/Aepyceros*	*Ovis aries*
		LC_138	size 2/3 bovid	*Ovis aries*
		LC_139	size 2/3 bovid	*Ovis aries*
		LC_149	caprine	*Antidorcas marsupialis*
		LC_176	caprine	*Antidorcas marsupialis*

**Figure 3.**
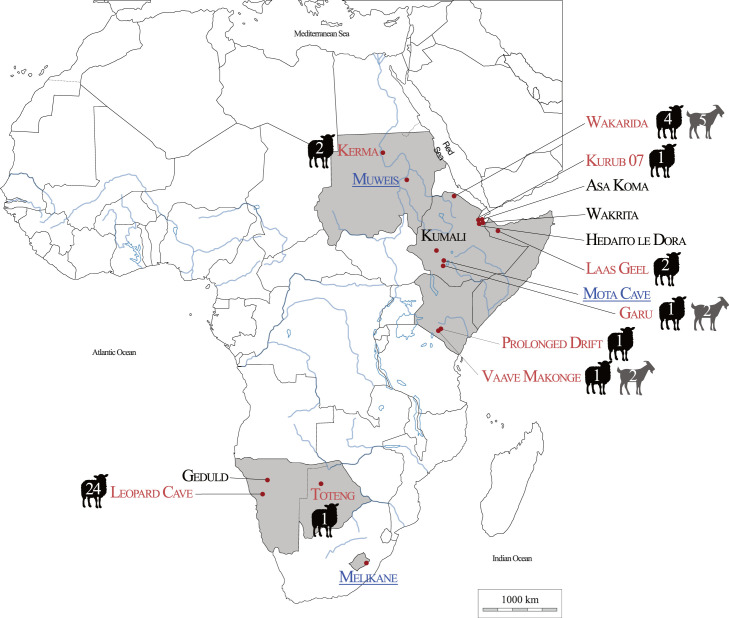
Synthesis of the taxonomic identifications obtained using palaeoproteomics. Sites in red yielded domestic caprines with numbers indicating the number of remains identified. Sites in blue and underlined indicate that palaeoproteomics analyses allowed species or genus identification only of wild antelope species. Sites in black indicate the lack of preserved proteins. Map modified after Lesur [7].

Our results show the presence of domesticated caprines at nine sites (seven in eastern Africa, two in southern Africa), represented in red in figure 3. In eastern Africa, one specimen was identified as sheep at Kurub Bahari Plain, Garu, Prolonged Drift and Vaave Makonge; two at Kerma, Laas Geel and four at Wakarida, along with two goats at Vaave Makonge and Garu and five at Wakarida. In southern Africa, one bone element was identified as sheep at Toteng and 24 at Leopard Cave (table 1 and figure 3). Ultimately, three archaeological sites (Muweis in Sudan, Mota Cave in Ethiopia and Melikane in Lesotho) presented specimens belonging to various wild bovid species. At Muweis, the preservation of the organic phase of the remains did not allow us to obtain identifications below the family level. At Mota Cave, one gazelle from genus *Nanger* and one bohor reedbuck (*R. redunca*) were identified, out of the four specimens analysed. At Melikane, of the 19 analysed specimens, 18 were identified as belonging to the rhebok, *P. capreolus*, whereas the last specimen from the site provided the protein signature of a klipspringer (*O. oreotragus*).

### 4.3. Direct radiocarbon dating of molecularly identified sheep and goats

To ascertain the antiquity of molecularly identified domesticates, some remains were directly radiocarbon dated. One goat from Garu, a sheep and a goat from Wakarida, the two sheep from Laas Geel, two goats and one sheep from Vaave Makonge, one sheep from Prolonged Drift, the previously reported sheep from Toteng [58], as well as two sheep from Leopard Cave [34] were directly radiocarbon dated (table 2). Overall, C/N ratios were good, although collagen yields differ between sites (electronic supplementary material, table S4). In eastern Africa, the dates obtained for the domesticates range from 3143–1198 cal. BP for the Kenyan samples to 259–30 cal. BP for the sample of Garu (Ethiopia). Those from southern African sites range from 1343–2863 cal. BP for the sample of Toteng to 919–678 cal. BP for the samples of Leopard Cave in Namibia (table 2). We do not present any radiocarbon dates for four other sites that yielded domesticated remains for several reasons. At Kerma, the analysis of one sample did not allow us to obtain any date because not enough material was extracted to allow dating. Finally, the sample from Kurub Bahari plain was modern (post-bomb), and thus excluded from the discussion.

**Table 2.** Radiocarbon dates obtained for the specimens identified as domesticates. Ages BP were calibrated online using OxCal 4.4 [77] and calibration curves IntCal20 for Garu, Wakarida, Kurub Bahari Plain and Laas Geel [78] and SHCal20 for Vaave Makonge, Prolonged Drift, Toteng and Leopard Cave [79].

sample code	AMS number	site (country)	species (molecular ID)	age BP	uncertainty	calibration (2*σ*, 95.4%)
GAR_91	ECHo_2997	Garu (Ethiopia)	*C. hircus*	86	27	259–30 cal. BP
WAK_34	ECHo_2996	Wakarida (Ethiopia)	*C. hircus*	677	28	674–561 cal. BP
WAK_41	ECHo_2995		*O. aries*	1643	28	1684–1412 cal. BP
KUR_58	ECHo_2990	Kurub Bahari plain (Ethiopia)	*O. aries*	−628	25	Post-bomb (modern)
LG_61	ECHo_2988	Laas Geel (Somaliland)	*O. aries*	251	25	427–151 cal. BP
LG_62	ECHo_2989		*O. aries*	1461	34	1390–1300 cal. BP
GvJm_65	ECHo_2991	Vaave Makonge (Kenya)	*C. hircus*	2257	29	2332–2134 cal. BP
GvJm_66	ECHo_2992		*O. aries*	2133	30	2287–1998 cal. BP
GvJm_67	ECHo_2994		*C. hircus*	2226	29	2325–2096 cal. BP
GrJi1_165	ECHo_4135	Prolonged Drift (Kenya)	*O. aries*	2898	32	3143–2863 cal. BP
TOT_14	ECHo_2769	Toteng (Botswana)	*O. aries*	1410	30	1346–1178 cal. BP
LC_131	ECHo_2770	Leopard Cave (Namibia)	*O. aries*	871	30	790–678 cal. BP
LC_135	ECHo_2768		*O. aries*	959	30	919–743 cal. BP

## 5. Discussion

### 5.1. Collagen type I sequence references of African wild bovid species

Out of the 37 samples that provided informative proteomic profiles, only one sample did not yield an exploitable protein sequence, indicative of an overall rather good organic preservation. The peptide markers identified show a higher variability of COL1A2 than of COL1A1. Interestingly, the taxonomic markers are located on other peptides than the ones used for distinguishing the domesticated caprines, meaning that the peptide combinations described in this paper can be used for distinguishing between wild and domestic bovids. Analysed Antilopinae do not present many variations among themselves, except for two species: the springbok *A. marsupialis* and the klipspringer *O. oreotragus*, with two and three specific peptides on COL1A2, respectively. One peptide on COL1A2 is conserved in all groups, except for the wild and domestic Caprinae species, indicating that the subfamily does present SAPs that are not shared by the other taxa. The COL1A1 peptide previously described for sheep identification, 757 … **A**GEVGPPGPPGPAGEK … 772, is not shared with any other species, thus making it highly valuable for species distinction of sheep in archaeological assemblages [58]. The COL1A2 peptide of goat is only present in the other *Capra* species, such as *C. nubiana*, as previously reported [80]. All other peptides presenting SAPs are alternatively shared between the 19 species and the other bovids, cattle and zebu, or the caprines, sheep and goat, demonstrating a strong conservation within type I collagen sequence in family Bovidae. These results are in accordance with previous genetic or proteomic studies [18,39,43], and generally reflect mammalian biology.

### 5.2. Domesticated sheep and goats presence in eastern and southern Africa between 3200 and 700 cal. BP

The presence of domesticated caprines is confirmed by this study at seven sites from eastern Africa and two from southern Africa. Only three sites (Wakarida, Garu and Vaave Makonge) yielded remains attributed to both sheep and goat. Sheep predominate in the assemblages analysed here: among the 47 molecularly identified domesticates, 38 belong to sheep. These numbers are likely to be inflated by the 24 sheep remains from Leopard Cave [34]: most elements sampled are probably from the same individual based on the use of various bone elements. Despite this bias, our results support the widespread archaeological view that *O. aries* is the dominant domesticated species at early sites [17,81–83], in spite of previous cautions on morphological identification of the two caprine species [32].

Although African breeds of the two species adapt well to harsh environments, sheep and goats have different dietary and environmental preferences and are often managed with different goals [84]. The predominance of *Ovis* over *Capra* in sampled archaeological assemblages is surprising, because they are often herded together in more recent times. Southwest Asian fat-tailed sheep have unique physiological traits, such as being able to store energy and to survive and reproduce in times of low food intake [85,86]. Muigai and Hanotte argue that the breed should have been widely distributed and valued for its adaptive capacities [20]. Individual fat-tailed sheep would also have offered foragers just beginning to keep caprines while still relying on the lean meat of hunted wild bovids a considerable yield of physiologically vital fats [87]. The presence of fat-tailed sheep breeds in both eastern Africa (Adal in Ethiopia, Somali in Somalia, Ethiopia, Djibouti and Kenya, Red Maasai in Kenya, Tanzania and Uganda, and Blackhead Persian in Somaliland) and southern Africa (Damara in Namibia and Afrikaner, Zulu, Van Rooy and Meatmaster in South Africa) suggests that this breed was successful in spreading widely across the continent. However, we should note that the sheep from Kerma have been described as ‘long-legged hairy’ and thin-tailed, with fewer caudal vertebrae than other thin-tailed sheep from Ethiopia, something that Chaix interprets as a primitive feature inherited from the mouflon [88–90]. Representations of thin-tailed sheep at Beni Hassan in Khnumhotep II's tomb during the Egyptian XIIth dynasty also attest to the presence of these animals in the northeastern part of Africa [91]. Thus, both breed types spread widely. Which specific sheep breed was present during the early steps of the diffusion of herding in Africa cannot, for now, be resolved using palaeoproteomics data. This is due to the high degree of homology in the COL1 sequence within the Bovidae family overall. However, considering the fast development of the field, along with further developments of mass spectrometry techniques, we believe that it will be possible to tackle this question in the near future.

The palaeoproteomics species identifications in this study and in previous ones [18,34,48,49,54,58] provide valuable new data points with which to assess different chronological models proposed for caprines dispersals from eastern to southern Africa [17]. Possible routes that domestic caprines and/or associated herders could have taken to reach the southernmost tip of Africa remain debatable. Some of the bones we identified as caprines provided the expected chronological age, based on earlier dating of the sites from which they originate, but many of them yielded ^14^C dates that were more recent than the ones proposed in the literature for the associated archaeological layers. For example, at Laas Geel, the radiocarbon dates obtained in our study indicate the presence of domestic sheep only *ca* 1400 cal. BP. Indeed, the Holocene layers where the identified sheep remains come from were previously dated to *ca* 4800 BP [92]. However, the upper layers of shelter 7 appear to have been reworked during more recent occupations with the presence of a rectangular pit lined with granite slabs, which could not be dated but seems to have penetrated earlier Holocene levels. In southern Africa, the dates obtained at Leopard Cave (919–678 cal. BP) and Toteng (1346–1178 cal. BP), are more recent than the date of *ca* 2000 BP described for the arrival of caprines in the region [17,18,55]. As demonstrated by Sealy and Yates, the mobility of sheep remains (and most probably of other materials) through rock shelter deposits during the site formation alters the overall comprehension of the archaeological context [93]. More systematic geoarchaeological work thus needs to be performed to refine the overall understanding of the site.

As described by Sadr [17], three scenarios are currently proposed for the spread of domesticated caprines based on archaeological, linguistic and genetic data: the migration model, in which the caprine herds were brought south by mass movement of Khoe-speaking groups [29,94,95]; the demic model, in which livestock was traded from one group to a neighbouring one, without human migration [96]; and finally, a ‘small-scale’ model in which their diffusion was more sporadic, as hunter–gatherer groups slowly became ‘hunters with sheep’ [4,97]. Although not intended to document the *first occurrences* of domesticated caprines in both eastern and southern Africa, our dataset, combined with the available zooarchaeological evidence in the same archaeological layers for each site, allows us to observe the associated presence of wild species of antelope and domesticated caprines at all studied sites. These data suggest a mixed subsistence economy, with a continuity of hunting associated with the reliability of herding, something consistent with all three of the scenarios described by Sadr.

**Figure 4.**
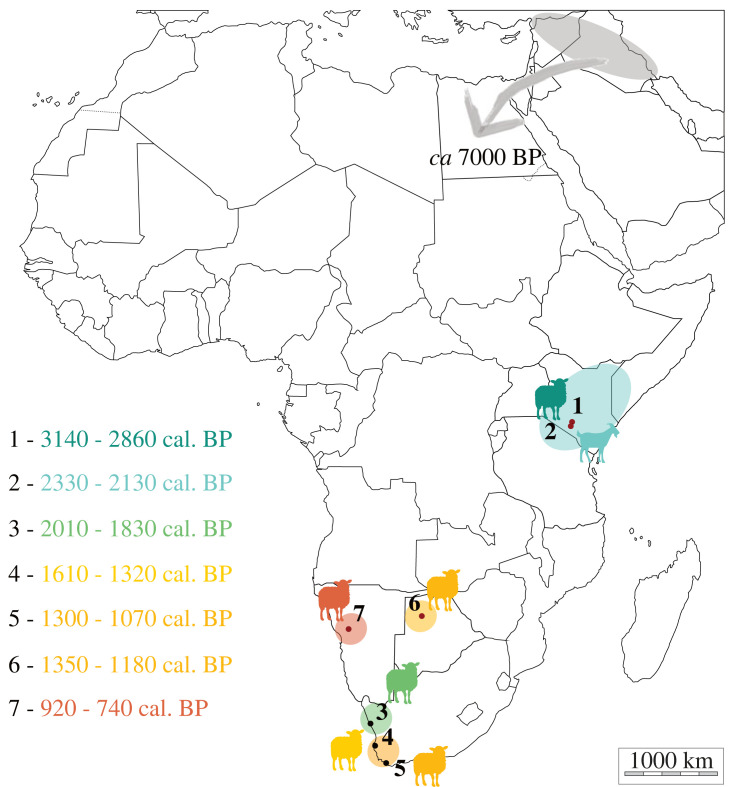
Geographical chronological scenario of early caprines diffusion since their first introduction in the northern part of Africa, solely based on directly dated remains of either sheep or goat which were molecularly identified. Data taken from this study (red dots) and Coutu *et al*. ([18], black dots). Archaeological sites are: 1, Prolonged Drift; 2, Vaave Makonge; 3, Spoegrivier; 4, Kasteelberg; 5, Die Kelders; 6, Toteng; and 7, Leopard Cave. Dates correspond to calibrated radiocarbon dates and are represented using a colour gradient from cooler to warmer to reflect their antiquity (with warmer colour being the more recent evidence).

Our conclusions on species attributions, coupled to the direct radiocarbon dating of molecularly identified remains of sheep and goat, indicate with certainty the presence of caprines from the Later Stone Age in both eastern and southern Africa (figure 4). Based on the data we present here, domesticated caprines are dated to 3143–2863 cal. BP at Prolonged Drift, their presence at Toteng is dated to 1346–1178 cal. BP, and they finally appear in the faunal assemblage of Leopard Cave around 919–743 cal. BP. Regardless of the scenario, the majority of southern African sites seem to support ‘hunters with sheep', rather than the immigration of sheep-herding groups. The pattern of diffusion described above appears to correspond to the dispersal route of domestic caprines through Africa, following the path of a tsetse-free corridor, and highlights the significant time lag in the presence of domesticates between the two regions [17,21]. There is also a consistency between the data presented here and the ones from the available literature on molecularly identified and directly dated domesticated caprine remains [18]. Future palaeoproteomics and radiocarbon analyses exploring other important locations and areas, including those in Zimbabwe (such as Bambata Cave [98]) and Mozambique will refine our understanding of the speed and paths that sheep and goats took from northeastern to southern Africa; simply because most of our existing data come from the western third of the region does not mean that caprines may not also have spread along more easterly routes [99,100]. Finally, and even though we acknowledge the strong biases of our dataset (low number of sites, selection of faunal remains that may correspond to caprines, few assumed early occurrences), our palaeoproteomics analysis coupled with direct dating of domesticated caprines helps to refine the history of herding in eastern and southern Africa. We believe that this methodological approach offers very exciting new perspectives for documenting the interactions of past African populations with their environment.

## Data Availability

All data associated with modern reference samples, as long as the ones for the archaeological samples can be found in the electronic supplementary material, information of this paper. Raw proteomics data for both modern references and archaeological specimens have been deposited to the ProteomeXchange Consortium via the PRIDE partner repository [101], with dataset identifier PXD045452. Each file deposited follows the nomenclature used in the present paper. The fasta file containing the COL1 sequences reference database of the wild bovids species can be downloaded from Dryad using the following link https://doi.org/10.5061/dryad.xd2547dnw [102]. Supplementary material is available online [103].
